# Maternal Anxiety Associated with Nocturnal Childhood Enuresis

**DOI:** 10.3390/children9081232

**Published:** 2022-08-15

**Authors:** Javier Quiroz-Guerrero, Alejandra Ortega-Pardo, Rafael Edgardo Maldonado-Valadez, Raúl García-Díaz de León, Lorena Mercado-Villareal, Edel Rafael Rodea-Montero

**Affiliations:** 1 Department of Pediatric Urology, Hospital Regional de Alta Especialidad del Bajío, Leon 37660, Mexico; 2 Department of Pediatric, Hospital Regional de Alta Especialidad del Bajío, Leon 37660, Mexico; 3 Department of Urology, Hospital Regional de Alta Especialidad del Bajío, Leon 37660, Mexico; 4 Department of Psychology, Hospital Regional de Alta Especialidad del Bajío, Leon 37660, Mexico; 5 Department of Research, Hospital Regional de Alta Especialidad del Bajío, Leon 37660, Mexico

**Keywords:** anxiety, children, mothers, nocturnal enuresis

## Abstract

Introduction: Nocturnal enuresis is a common problem affecting 20% of 5-year-old children; its prevalence decreases with age. Nocturnal bedwetting in children younger than 5 is generally accepted by parents and society, but the expectation of parents is that children will achieve a higher degree of responsibility and increased control with age. Some studies have identified maternal distress as a factor associated with childhood urinary incontinence; specifically, maternal stress, maternal depression, and maternal anxiety. The aim of this study was to compare the degree of anxiety (trait and state) among mothers of children with nocturnal enuresis and mothers of healthy children. Methods: This was a prospective, cross-sectional, comparative study including two groups: a control group of 25 mothers of healthy children from open population, and an enuresis group of 25 mothers of children with nocturnal enuresis of the pediatric urology clinic of a third-level Mexican Hospital. STAI-T and STAI-S tests were performed and assessed blindly. Quantitative variables were compared using the Mann–Whitney U test, and qualitative determinations using a chi-square test or Fischer’s exact test. Results: The STAI-S and STAI-T tests results identified 14 (56%) mothers of enuretic children with moderate-to-severe trait anxiety versus 4 (16%) mothers from the control group and moderate-to-severe state anxiety in 23 (92%) of the mothers of enuretic children compared to 7 (28%) control-group mothers. The anxiety scores were significantly higher for the enuresis group for both tests: STAI-T: 53.00 ± 8.39 versus 41.52 ± 9.61 (*p* < 0.001) and STAI-S: 56.48 ± 6.83 versus 43.84 ± 10.57 (*p* < 0.001). Conclusion: Mothers of children with nocturnal enuresis present state anxiety ranging from moderate to severe. In clinical practice, our results indicate that the transitory emotion experienced by mothers of enuretic children cannot be neglected in an enuresis treatment program, state anxiety needs to be evaluated, and psychological interventions need to be implemented.

## 1. Introduction

Nocturnal enuresis, identified as R32 according to International Classification Diseases (ICD-10) [1], is a common problem affecting 20% of 5-year-old children; its prevalence decreases with age [2,3]. Although nocturnal enuresis is easily noticed by parents and physicians, its treatment requires a complex approach [4] due to the condition involving a complex interrelationship of biological, genetic, and psychosocial factors [5]. There is little public awareness of enuresis being a treatable condition, and consequently many families choose to keep it a secret, expecting a spontaneous resolution, instead of seeking medical help [6]. Nocturnal bedwetting in children younger than 5 is generally accepted by parents and society [3], but the expectation of parents is that children will achieve a higher degree of responsibility and increased control with age [7]. When this does not happen, nocturnal enuresis becomes a source of stress for the child and the family [8], and it often has a significant negative impact on the child’s self-image and development [7,9,10]. The risk of waiting is that some parents become intolerant with their children wetting the bed [11,12,13].

Most studies published in the literature on nocturnal childhood enuresis focus on the determination of the prevalence of the pathology in different ethnic groups [14,15,16,17], on the identification of related factors [18,19,20], on the medical treatment of the organic problem of the children [21,22,23], and/or on assessing the psychological impact of this condition on the children [24,25,26] rather than on their families. Mothers of enuretic children are generally viewed as the primary caregiver. After each enuresis episode, the mothers take care of the cleaning, the laundry, and the prevention of skin diseases, as well as the control of the child’s reactions and those of other members of the family [27]. Parents in general, but particularly mothers, report more behavioral problems from enuretic children; mothers also tend to present higher degrees of stress. Parent stress levels correlate highly with enuretic children’s behavioral problems [28].

There are studies about the prevalence of nocturnal enuresis among children and its association with the mental health of mothers [29], about maternal attributions and tolerance for nocturnal enuresis [7], and about the quality of life and stress in mothers of children with enuresis [30]. Other studies have identified maternal distress as a factor associated with childhood urinary incontinence [31]; specifically, maternal stress [27], maternal depression, and maternal anxiety [5]. Typically, anxiety symptoms are evaluated in terms of state and trait anxiety [30]. Spielberger defined trait anxiety as an individual’s predisposition to respond to typical everyday situations, such as feelings of stress, worry, and discomfort. State anxiety is defined as a transitory emotion experienced by a subject in a specific situation characterized by physiological arousal and consciously perceived feelings of apprehension, dread, and tension [32]. Increased anxiety levels have been reported in parents of enuretic children [33,34]. In addition, Durmaz et al. identified that mothers of children with nocturnal enuresis also show higher rates of anxiety than those of the typically developing children [35].

It is also important to evaluate the anxiety in the mothers of enuretic children and to establish the degree of this maternal distress problem developed by the mothers in order to identify if psychological support could be needed for a more functional anxiety management related to nocturnal enuresis. 

In this study, we focused on anxiety symptoms in terms of state and trait anxiety, not on anxiety disorders per se. The aim of this study was to assess and compare the degrees of anxiety (trait and state) in mothers of healthy children (control group) and mothers of children suffering from nocturnal enuresis (enuresis group). We hypothesized that mothers of enuretic children show higher state anxiety than typically developing children because of the maternal distress generated by the enuresis problem.

## 2. Materials and Methods

We conducted a prospective, cross-sectional, comparative study with a sample of Mexican mothers of children with enuresis and Mexican mothers of healthy children that were recruited between August 2010 and September 2020. This study included mothers of healthy children (older than 5 years and younger than 16) that were enrolled from open population (Group I: Control) and mothers of patients (children older than 5 years and younger than 16) with enuresis (at least 5 days of the week with nocturnal bedwetting) (Group II: Enuresis) of the Pediatric Urology Clinic of a third-level care Mexican hospital: Hospital Regional de Alta Especialidad del Bajío (HRAEB), located in León City in Guanajuato state (Mexico). 

In both groups, data regarding mothers (age, education, marital status, and employment status) and regarding children (age, sex, and number of siblings) were obtained. All mothers included were literate and capable of answering the complete State Trait Anxiety Inventory (STAI), which is made up of two self-assessment scales designed for measuring anxiety as a trait (STAI-T) and as a state (STAI-S) [36,37]. The exclusion criteria for both groups were providing care for an elderly, chronically ill, or disabled relative; caring for another child younger than 3 years; having another child at home who needs special health care; or history of chronic illness, pulmonary disease, cardiovascular disease, or psychiatric disorder. Informed written consent was obtained from all mothers involved in the study upon enrolment; each mother was informed about the study and signed a written consent form when they agreed to enroll.

The study was carried out in compliance with the Declaration of Helsinki (Fortaleza-Brazil, 2013) [38], the ICH Harmonized Tripartite Guideline for Good Clinical Practice, and the regulations in Mexican general health law, chapters I and V, regarding ethical aspects of research in humans [39]. The study protocol was reviewed and approved by the Research and Ethics Committee of the HRAEB (protocol code CI-HRAEB 004-09 and date of approval 1 August 2009).

The independent variables were enuretic child and healthy child, and outcome variables were the degrees of maternal trait and state anxiety (STAI-T and STAI-S). The STAI-T scale measures the frequency of anxiety symptoms, and the STAI-S measures the anxiety symptoms experienced by a subject in a specific situation. Both scales include 20 Likert-type items and have been shown to have high reliability (values of Cronbach alpha between 0.52 and 0.8). Each instrument can score between 20 and 80 points, divided into the following four degrees of anxiety: absent (20 to 39), mild (40 to 49), moderate (50 to 59), and severe (60 to 80). Both STAI-T and STAI-S inventories were administered to mothers of children with enuresis of the Pediatric Urology clinic of HRAEB and to the control group mothers in the open population. The questionnaires were blindly assessed and marked at the HRAEB Clinical Psychology Department.

### 2.1. Sample Size

The calculated sample size was n=50 mothers, divided into two groups of 25 each. Based on the calculated sample size, $p_{1}=0.5$ and $p_{2}=0.9$ were established as the probabilities that a mother would suffer or not suffer anxiety, respectively, with α=0.05 and β=0.20 (power) at a 95% confidence interval. The calculation was performed with the Stanton A program Primer Biostatistics V.04. Sample selection was non-probabilistic, by convenience.

### 2.2. Statistical Analysis

All the data were analyzed blindly using the statistical software R version 3.6.0 [40]. Descriptive statistics were used to analyze the qualitative and quantitative characteristics of the children, mothers, and fathers. Quantitative variables were compared between groups using the Mann–Whitney U test [41], and qualitative determinations were compared using a chi-square test or Fischer’s exact test. A cross-sectional comparison of the mothers’ STAI-T and STAI-S (degree of trait and state anxiety) mean scores between the groups was performed using the Mann–Whitney U test [41]. In all cases, 95% confidence intervals were applied, and in all tests, a statistical significance level of α=0.05 was set.

## 3. Results

The study included two groups, a control group of 25 mothers of healthy children from open population, and an enuresis group of 25 mothers of children with nocturnal enuresis of the Pediatric Urology Clinic of a third-level Mexican hospital. The control group was composed of mothers of 12 females and 13 males, and the enuresis group of mothers of 11 females and 14 males, with no significant difference in gender ratios between the two groups. The means and standard deviations of the children’s ages were 7.04 ± 1.95 in the control group and 6.68 ± 1.84 (*p* = 0.462) in the enuresis group, reflecting homogeneous groups regarding age. There was significant difference for number of siblings (*p* = 0.047) between the groups, with a slightly larger value in the enuresis group.

Mother age was significantly older in the enuresis group (35.52 ± 5.77) compared with control group (31.96 ± 4.00), *p* = 0.016. There were 19 (76%) married mothers in the control group and 23 (92%) in the enuresis group, with no significant difference (*p* = 0.247). There was a significant difference in number of years of education between the groups, with the control group being higher (14.64 ± 2.06, and 12.6 ± 3.74, *p* = 0.049). Employment status contained 21 (84%) actively employed mothers in the control group and 12 (48%) in the enuresis group (*p* = 0.016), which was a significant difference. Regarding father employment status, no significant difference was detected between the groups (Table 1).

**Figure 1:**
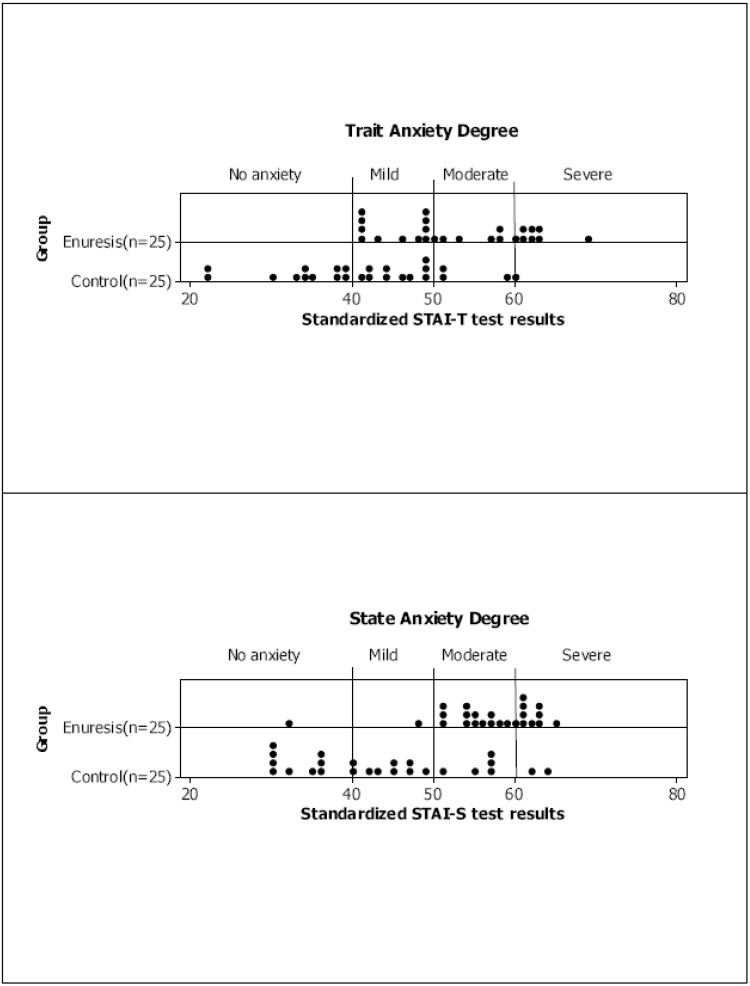
Representation of the mothers’ degree of anxiety based on the standardized STAI-T and STAI-S test results per group.

**Figure 2:**
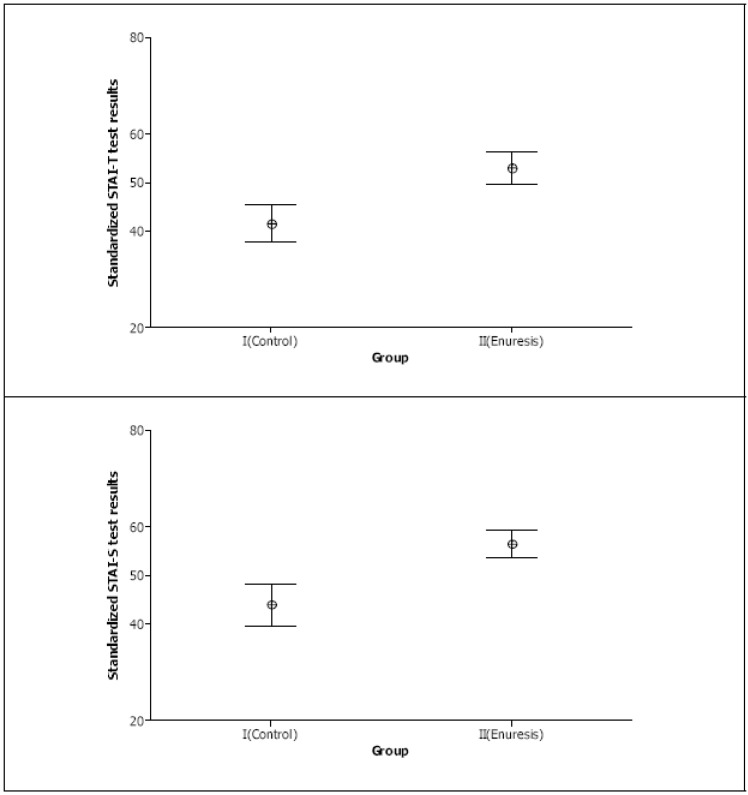
Standardized STAI-T and STAI-S test results. Group means are shown with 95% confidence intervals.

Mother STAI-T and STAI-S test scores are presented in Figure 1 by group and degree of anxiety. In Table 2, it can be seen that 14 (56%) mothers of children with enuresis showed trait anxiety between moderate and severe versus 4 (16%) mothers in the control group; a degree of state anxiety ranging between moderate and severe was observed in 23 (92%) mothers of children with enuresis compared with 7 (28%) mothers of control children. The non-parametric Mann–Whitney U test was used to compare the anxiety scores from the control and enuresis groups. The anxiety scores were significantly higher for the enuresis group for both tests: STAI-T, 53.00 ± 8.39 versus 41.52 ± 9.61 (*p* < 0.001) and STAI-S, 56.48 ± 6.83 versus 43.84 ± 10.57 (*p* < 0.001) (Table 3). Finally, 95% confidence intervals were constructed for the score means from each group (Figure 2).

## 4. Discussion

In our study, we investigated anxiety in mothers who have a child with nocturnal enuresis. Therefore, this work, unlike most studies on pediatric nocturnal enuresis, enables a comparison of the degrees of trait and state anxiety in mothers of children suffering from nocturnal enuresis and mothers of healthy children.

In the present study, the results showed significantly higher anxiety scores in both scales (trait and state) for 25 mothers in enuresis group compared with 25 mothers in control group. We found that 56% of mothers of children with enuresis showed trait anxiety between moderate and severe, versus 16% of mothers in the control group, and that 92% of mothers of children with enuresis showed state anxiety between moderate and severe, versus 28% of mother in the control group. We observed results similar to those described by Hussong et al. [42], who identified higher state and trait anxiety in 40 parents (36 mothers) of enuretic children compared with 40 parents (37 mothers) in control group. In concordance, Naitoh et al. [43] also found a significantly higher state anxiety in 139 mothers of enuretic children compared with 109 mothers in control group.

In contrast with our findings, Egemen et al. [30] did not find a significant difference in trait or state anxiety when compared STAI results of 28 mothers of enuretic children vs. STAI results of 38 mothers of healthy children. However, they reported that trait and state anxiety scores were above 40 in the study group, which shows a high anxiety score of mothers of enuretic children. 

In our results, the degree of state anxiety in mothers of children with enuresis ranged between moderate and severe. We identified a learned and maintained anxious reaction in mothers, who would react to a child wetting the bed with intolerance, anguish, or concern, manifesting a significant degree of state anxiety. Butler et al. [7] assessed mothers’ tolerance to children’s nocturnal enuresis problems and identified that generally, mothers perceived the causes of enuresis as persistent and uncontrollable, often blaming the child for the lack of control. Especially considering that mothers are generally the main protagonists of their children’s care as primary caregiver, a tense and anxious atmosphere in the family can be reflected in the child’s behavioral development, since the family is the first psychosocial environment influencing the child’s development and behavior [44]. In view of the results of the present study, we propose the need for a comprehensive approach to the treatment of nocturnal enuresis, which should consider, in addition to treating the organic problem of the children, assessment and psychological treatment of the mother, as patient, with the aim to avoid psychosocial damage.

This study has certain limitations. First, this study was cross-sectional in nature, and therefore, causality cannot be inferred. Second, the study sample consisted only of Mexican mothers, which may limit generalizability to other ethnic groups. Third, the findings are based on a very small number (*n* = 25) of mothers of children with nocturnal enuresis, thus interpretation should be careful. Fourth, stressful life events could explain both children’s enuresis and maternal anxiety. Fifth, unfortunately, no information was available on the family’s circumstances or on the child’s emotional and behavioral function. Last, confounding factors, such a nutritional status, stress, burnout, and other relevant conditions (smoking and alcohol consumption) of the mothers, must be considered as possible contributors to the anxiety and could be controlled in a more comprehensive way. However, the strengths of this study include its prospective design, the use of standardized instruments, and the focus on state anxiety.

In general, despite maternal distress being identified as a factor associated with childhood incontinency [31], the evaluation of maternal stress, maternal depression, and maternal anxiety is not part of the clinical care and follow up of the mothers of enuretic children. There is a need for an increased understanding of the role of the maternal psychological state as a factor associated with childhood urinary incontinence, and further research is needed to determine whether these associations are likely to be causal [5]. Finally, longitudinal prospective studies to characterize the progression of the distress among mothers of enuretic children are also necessary.

## 5. Conclusions

Our study suggests that mothers of children with nocturnal enuresis present state anxiety ranging from moderate to severe. Our findings would help to provide timely psychological care to mothers of enuretic children and to avoid psychosocial damage. It is important to pay attention to anxiety disorders in mothers of enuretic children in order to design more tailored approaches to treatment, clinical care, and follow-up of these mothers. In clinical practice, our results indicate that the transitory emotion experienced by mothers of enuretic children cannot be neglected in an enuresis treatment program, state anxiety needs to be evaluated, and psychological interventions need to be implemented.

## Figures and Tables

**Table 1:** Description of the child, mother, and father characteristics in each group.

		Group I (Control) *n* = 25	Group II (Enuresis) *n* = 25	Comparison between Groups
Children				
	Child age (years)	7.04 (1.95)	6.68 (1.84)	U = 275.50, *p* = 0.462
	Female, n (%)	12 (48%)	11 (44%)	X^2^ = 0.0805, *p* = 0.777
	Male, n (%)	13 (52%)	14 (56%)	X^2^ = 0.0805, *p* = 0.777
	Number of siblings	0.76 (0.78)	1.24 (0.83)	U = 216.50, *p* = 0.047 *
Mothers				
	Mother age (years)	31.96 (4.00)	35.52 (5.77)	U = 188.50, *p* = 0.016 *
	Married, n (%)	19 (76%)	23 (92%)	*p* = 0.247 ^a^
	Education (years)	14.64 (2.06)	12.6 (3.74)	U = 220.50, *p* = 0.049 *
	Employed, n (%)	21 (84%)	12 (48%)	*p* = 0.016 * ^a^
Fathers				
	Employed, n (%)	22 (88%)	22 (88%)	*p* = 0.999 ^a^

Values are given as means (standard deviations). ^a^ Fisher’s exact test. * Significant *p*-value.

**Table 2:** Trait anxiety and state anxiety frequencies in each group.

	Group I (Control) *n* = 25		Group II (Enuresis) *n* = 25	
Anxiety Degree	Trait	State	Trait	State
No anxiety (20–39) *	11	9	0	1
Mild (40–49) *	10	9	11	1
Moderate (50–59) *	3	5	6	13
Severe (60–80) *	1	2	8	10

* Classification based on STAI standardized score scales.

**Table 3:** Description of the mothers’ standardized STAI tests by group.

Variable	Group I (Control) *n* = 25	Group II (Enuresis) *n* = 25	Comparison between Groups
STAI-T (score 20–80)	41.52 (9.61)	53.00 (8.39)	U = 118.00, *p* < 0.001 *
STAI-S (score 20–80)	43.84 (10.57)	56.48 (6.83)	U = 107.50, *p* < 0.001 *

Values are given as means (standard deviations). * Significant *p*-value.

## Data Availability

All data underlying the findings are available on request to the corresponding author (edel.rodea@hraeb.gob.mx).
